# A case of dystrophic calcification in the masseter muscle

**DOI:** 10.1186/s40902-017-0130-4

**Published:** 2017-11-05

**Authors:** Heon-Young Kim, Jung-Hyun Park, Jun-Bum Lee, Sun-Jong Kim

**Affiliations:** Department of Oral and Maxillofacial surgery, Ewha Womans University Mok-dong Hospital, Mok 5-dong, Yangcheon-gu, Seoul, 158-710 South Korea

**Keywords:** Masseter muscle, Dystrophic calcification, Pathologic soft tissue calcification, Trauma

## Abstract

**Background:**

Dystrophic calcification can occur in any soft tissue with the absence of a systemic mineral imbalance and is often associated with trauma, infection, or inflammation. It is easily found in the site of the heart and skeletal muscles and rarely appears in the head and neck area.

**Case report:**

We present a rare case of multiple calcified masses in the left masseter muscle of a 26-year-old female with a history of trauma in the area. In computed tomography, multiple radiopaque masses were observed inside the left masseter muscle and blood test results were normal. The calcified masses were diagnosed as dystrophic calcification and removed by surgery without any complications.

**Conclusion:**

Different types of calcifications may occur in the cheek area, and they need to be distinguished from dystrophic calcification. Thorough clinical examination and history taking is required together with blood testing and radiographic examinations.

## Background

Pathologic soft tissue calcification of the cheek is an uncommon condition. There are many different types of calcifications, which includes dystrophic calcification, metastatic calcification, phleboliths, myositis ossificans, calcifications within lymph nodes, and calcified cutaneous aces, making differential diagnosis difficult [1]. In order to manage these lesions, they need to be distinguished from others that occur at the same area. Precise examination and selection of appropriate imaging, such as plain radiographs, ultrasonography, computed tomography (CT) with contrast, and magnetic resonance imaging (MRI), are important to aid in differentiation. Histological evaluation is also essential to arrive at a final diagnosis [1].

Among many different types of calcification, a dystrophic calcification is deposition of calcium salt in the soft tissue which associated with trauma, infection, or inflammation without elevated serum calcium level [2]. The precise mechanism of the disease is unknown, but it seems to be related to necrosis and apoptosis of the tissue [3, 4]. Dystrophic calcification is most often seen in the heart muscles and valves and rarely appears in the head and neck area [5]. Currently, there is no established protocol for its treatment. Some clinicians have recommended observation, but others have suggested the surgical treatment case by case [6]. This article presents a rare case of the multiple dystrophic calcifications in the masseter muscle.

## Case presentation

**Fig. 1:**
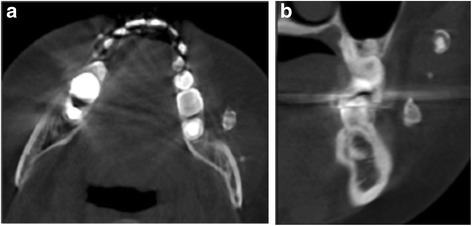
Preoperative computed tomography imaging shows multiple radiopaque masses inside the left masseter muscle. **a** Axial. **b** Coronal

A 26-year-old female patient visited to the Department of Oral and Maxillofacial Surgery in April 2017, with the complaint of feeling something hard in her left cheek for a few years. She had unremarkable medical history aside from a history of trauma in the left masseteric area when she was 4 years old. On physical examinations, surface texture and color of the skin and mucosa were in normal range without swelling or tenderness. When palpated, well-defined, oval-shaped, and movable nodules in her left cheek, less than 1 cm × 1 cm in size, were identified. Panorama and CT examinations were performed, and multiple radiopaque masses were observed inside the left masseter muscle (Fig. 1). The function of facial nerve and salivary flow of Stensen’s duct were normal. No trismus and cervical lymphadenopathy were noted. Blood test results were also normal. Serum calcium level was 9.4 g/dL and serum phosphorus level was 3.9 g/dL which were within normal ranges. From the clinical and radiographic evaluation whilst considering a history of trauma, the calcified mass was diagnosed as dystrophic calcification, which is known to occur in soft tissues, commonly in those with a history of trauma and the absence of systemic mineral imbalance.

**Fig. 2:**
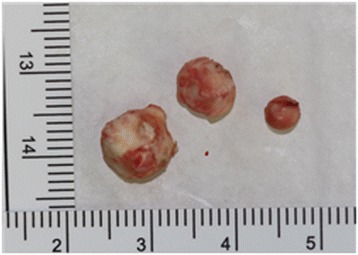
Excised calcified masses shows round- to oval-shaped and whitish-yellow-colored bodies of varying sizes

**Fig. 3:**
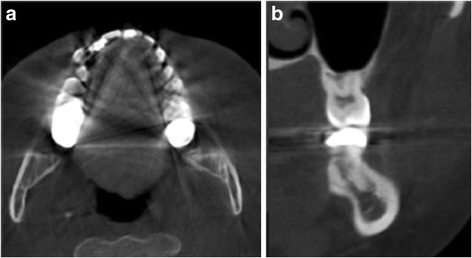
Postoperative computed tomography imaging shows that the calcified masses were excised completely. **a** Axial. **b** Coronal

**Fig. 4:**
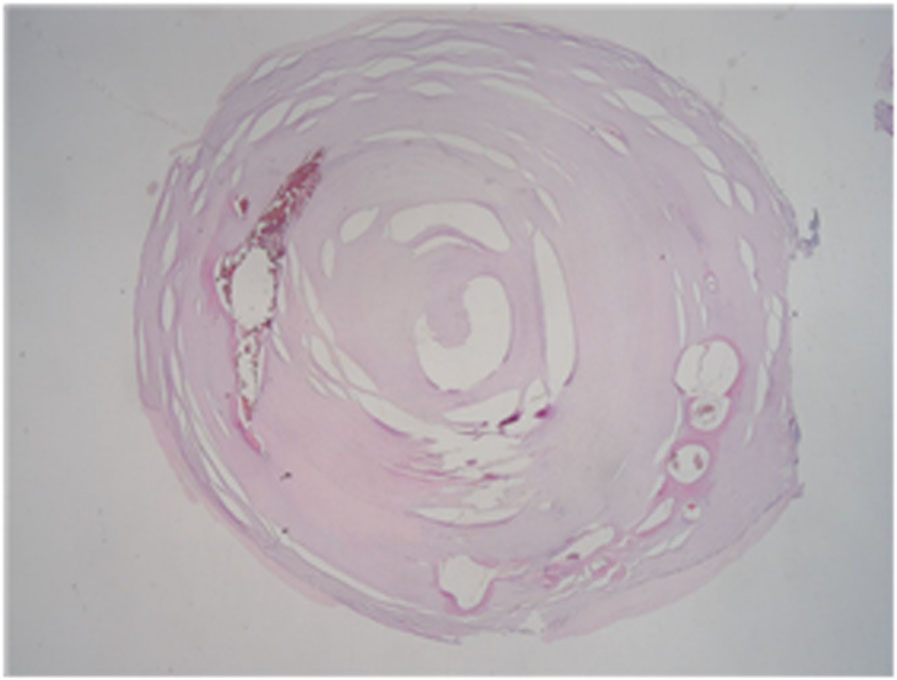
Microscopic examination revealed irregular multiple calcifications (×10)

Surgery was planned to remove the dystrophic calcification of the left masseter muscle. After intraoral incision in the left buccal mucosa, cautious dissection of masseter muscle was done. Muscle fibers of masseter were longitudinally separated to expose the calcified masses. They were firmly attached to the masseter muscle fibers and were bluntly separated from the muscle tissue. The three calcified masses, with the largest, having a size of 0.6 × 0.5 × 0.4 cm, presenting a round-oval shape and whitish-yellow in color, were removed with the attached muscle fibers (Fig. 2).After the calcified masses were excised completely, hemostasis was achieved and wound was sutured in layers. Postoperative recovery was uneventful with no damage to nearby structures such as nerves or Stensen’s duct. Postoperative CT imaging was taken, and complete removal of the calcified masses was confirmed (Fig. 3). Histopathological examination revealed microscopically ovoid hyalinized material with calcification in their center but could not be found fibrous tissue or vascular structure around the materials (Fig. 4).

### Discussion

There is a variety of conditions that may cause abnormal calcification in various tissues. It may have some connection with abnormal calcium phosphate metabolism as seen in metastatic calcifications [6]. However, dystrophic calcification is deposition of calcium salt in degenerated tissues related to normal calcium and phosphorous metabolism [7]. It is known that dystrophic calcification can occur in any soft tissue with the absence of a systemic mineral imbalance, easily found in the site of the heart muscle and skeletal muscle. It rarely appears in the gingiva, tongue, lymph nodes, and facial muscles and usually occurs in injured tissues [8, 9]. Based on the literature, our case is the fifth case of dystrophic calcification in a masseteric area to be reported (Table 1).

**Table 1:** Cases of dystrophic calcification in masseteric area have been reported

	Age/gender	Area	Treatment	Complication	Author	Year
1	21/M	Right masseter muscle (2 calcified masses)	Excision (extra-oral)	None	Sencimen M.	2010
2	14/M	Left masseter muscle (multiple calcified masses)	Excision (intra-oral)	None	Chetana S.	2012
3	17/M	Right masseter muscle (multiple calcified masses)	Excision (intra-oral)	None	S.A Mohiuddin.	2012
4	55/M	Right masseter muscle (1 calcified mass)	Observation	None	Sanjana R.	2017

The pathogenesis of dystrophic calcification is known to involve intracellular or extracellular initiation and propagation. Intracellular calcification is initiated with dead or dying cells that are not able to regulate intracellular calcium. After initiation, propagation of calcium phosphate crystalline formation occurs, which is affected by the concentration of Ca^2+^and PO^4−^ in the extracellular space [10]. Dystrophic calcification occurs when calcium is accumulated in the area of trauma or necrosis which may be caused by blunt trauma, inflammation, injections, and the presence of parasites [11].

In many cases, it appears early in childhood but it often tends to be diagnosed late since it shows no signs or symptoms. Therefore, it is found after lesion is enlarged enough to be palpated. In our case, we suppose that dystrophic calcification was caused by a trauma experienced at a young age. Because there had been no symptoms such as swelling or pain, it was discovered after a long period of time.

Different types of calcifications, including phleboliths, sialoliths, myositis ossificans, metastatic calcification, calcifications within lymph nodes, and calcified cutaneous aces, may occur in the cheek area, and they need to be distinguished from dystrophic calcification. Phleboliths are pathological, calcified thrombi that are associated with hemangiomas and developmental vascular malformations of the head and neck region [12]. Radiographically, they appear as round or oval radiopaque nodules, which may show a radiopaque center with surrounding onion ring-like concentric calcific rings [13]. In this case, there was no evidence of hemangioma or vascular malformation clinically and radiographically. Also, the calcified mass had no radiopaque center and concentric rings.

On the other hand, sialoliths are one of the most common diseases that appear in the salivary glands. Sialoliths of the parotid gland or duct need to be distinguished from calcification of the cheek, because the parotid gland is located behind the masseter muscle, with the Stensen’s duct, passing through it before opening into the oral cavity. Sialoliths may be composed of one or more stones and may cause pain or swelling when the salivary gland is stimulated by eating [13]. In our case, we ruled out a diagnosis of such because the calcified masses were located inside the masseter muscle, away from the Stensen’s duct or parotid gland based on the CT images. In addition, salivary flow of the duct was found to be normal.

Another differential diagnosis is myositis ossificans which results from trauma or heavy muscular strain associated with the bone or cartilage, producing reactive lesions. Clinically, it can be palpated beneath the skin or mucosa, as a minimally movable firm mass [14]. When the lesion is located within a muscle of mastication, it usually causes trismus because of limitation of the muscle [13]. On a radiograph, the linear streaks running in the same direction as the normal muscle fibers are regarded as a typical character for myositis ossificans [13]. In our case, the patient had no trismus and the radiographic finding was different from myositis ossificans [15].

In addition, calcifications within lymph nodes commonly involve cervical lymph nodes with metastatic deposits from malignancies such as squamous cell carcinoma and Hodgkin’s lymphoma. On a radiograph, they most often are irregular with a cauliflower appearance [13]. Metastatic calcifications occur due to increased calcium levels in the blood. Chronic renal failure, milk-alkali syndrome, extensive bone malignancy, and hypervitaminosis D are some of the conditions known to cause metastatic calcifications [1]. In our case, there was no evidence of malignancy causing metastasis or systemic mineral imbalance.

Due to this wide variety of diagnostic possibilities including phleboliths, sialoliths, myositis ossificans, calcification within lymph nodes, and the potential for malignancy, it is important to establish a proper diagnosis [4]. In fact, physiologic and pathologic soft tissue calcification of the head and neck is rare, and plain radiography is rarely helpful in diagnosing [16]. Therefore, thorough clinical examination and history taking is required together with blood testing and radiographic examinations such as CT, MRI, and ultrasound. In addition, fine needle aspiration for checking the contents of the lesions if deemed indicated can also help in confirming the diagnosis. The final diagnosis should be obtained after acquiring relevant information, and only then, the appropriate treatment can take place [17]. There is no established protocol for its treatment. Some clinicians have recommended observation. However, various factors such as the size and location of the lesion, and patient discomfort should be put into consideration. In such cases and if deemed necessary, meticulous surgical excision and periodic follow up are recommended [18, 19].

## Conclusions

Dystrophic calcification is deposition of calcium salt in degenerated tissues with the absence of a systemic mineral imbalance. It is often associated with trauma, infection, or inflammation and rarely appears in the head and neck area. Different types of calcifications, including phleboliths, sialoliths, myositis ossificans, metastatic calcification, calcifications within lymph nodes, and calcified cutaneous aces, may occur in the cheek area, and they need to be distinguished from dystrophic calcification. Thorough clinical examination and history taking is required together with blood testing and radiographic examinations such as CT, MRI and ultrasound.
